# Severe Cardiac Dysautonomia and Sudden Death in a Patient Presenting with Anginal Symptoms in Absence of Cardiovascular and Other Diseases: A Case Report

**DOI:** 10.31729/jnma.4828

**Published:** 2020-04-30

**Authors:** Rita Khadka, Rajiv Narang, Ashok Kumar Jaryal, Chetan Patel, Kishore Kumar Deepak

**Affiliations:** 1 Department of Basic and Clinical Physiology, B. P. Koirala Institute of Health Sciences, Dharan, Nepal; 2 Department of Cardiology, All India Institute of Medical Sciences, New Delhi, India; 3 Department of Physiology, All India Institute of Medical Sciences, New Delhi, India; 4 Department of Nuclear Medicine, All India Institute of Medical Sciences, New Delhi, India

**Keywords:** *angina*, *baroreflex*, *dysautonomia*, *heart rate*, *sudden death*

## Abstract

Angina is a type of chest pain, experienced by patients with ischemic heart diseases. Cardiac autonomic modulation as assessed by heart rate variability and baroreflex sensitivity is found reduced in ischemic heart disease patients. Marked reduction in heart rate variability and baroreflex sensitivity in ischemic heart disease patients is found associated with sudden cardiac death. We report a case of a 35-year-old man who presented with angina for the last few months. Thorough investigations showed no evidence of any cardiac or other systemic diseases. However, his cardiovascular autonomic modulation (as assessed by heart rate variability) and spontaneous baroreflex sensitivity were markedly reduced. The patient had sudden death within 6 months of follow-up. Reportedly, no other specific abnormalities were found before death. This case report suggests that patients presenting with typical chest pain as angina may have severe dysautonomia and risk of sudden death even in the absence of cardiovascular or any other known end-organ diseases.

## INTRODUCTION

Angina is a type of chest pain due to ischemia of the heart muscle. In patients with myocardial ischemia & myocardial infarction (MI), cardiac autonomic modulation as assessed with heart rate variability (HRV)^1^ is found reduced. Reduced HRV is one of the independent predictors of sudden cardiac death in MI.^2–4^ Baroreflex sensitivity (BRS) which assesses cardiovascular autonomic reflex integrity,^5,6^ is also found reduced in MI patients and found associated with sudden cardiac death.^2^ We report a case, presenting with angina had severe dysautonomia (marked reduction in HRV and BRS), and sudden death in the absence of cardiovascular or other diseases.

## CASE REPORT

A 35-year-old male visited cardiology Out Patient Department (OPD), All India Institute of Medical Sciences with precordial chest pain radiating to the left arm. He had breathlessness during the walk (New York Heart Association classification II) and reported fatigue even after a light work. He was a non-smoker and non-alcoholic. Since he presented as angina for the last few months, a thorough investigation was done for ischemic heart diseases (IHD).

On examination, resting HR, BP and respiratory rate were 100 beats/min, 98/70 mmHg, and 20 breaths/min respectively. His chest X-ray and 12-lead ECG were thoroughly studied. Both were normal. Routine investigation of blood were; Hb 10.2 gm/dl, TLC 6500, and ESR 40 mm 1st h, fasting blood glucose 90 mg/dl, blood urea 19 mg/dl, serum creatinine 0.9 mg/dl, serum sodium 136 mEq/l and potassium 4.2 mEq/l. Lipid profile of the patient was; total cholesterol 90 mg/dl, LDL 48 mg/dl, HDL 22 mg/dl, VLDL 20 mg/dl, and triglyceride 105 mg/dl. The patient had no symptoms of gastritis.

For treadmill test (TMT), Bruce protocol was followed: patients' total exercise time was 6 min 18 seconds, calculated oxygen consumption was 7.4 metabolic equivalent of task (MET), and HR reached 90% of targeted HR. There were no ST-segments elevation or depression or any other abnormalities observed in ECG during TMT, Rate pressure product was 22120 bpm*mmHg. Echocardiogram showed no structural or valvular abnormalities. The ejection fraction was 63%. The 24-hour Holter recording also showed no ST-segment elevation or depression or any arrhythmic changes. The phonocardiogram was normal. The stress Thallium-201 gated myocardial perfusion Single-photon emission computed tomography (SPECT) test showed no myocardial perfusion abnormality or any wall motion abnormality. His attitude towards life was positive as assessed with the WHO scoring system.^7^ He was put on aspirin 75 mg/day and oral nitrates because of clinical symptoms of angina.

There was a marked reduction in measures of shortterm HRV, and spontaneous BRS as assessed by sequence method (Table 1). Considering a severe degree of loss of cardiovascular autonomic tone, follow-up after every 3 months for the recording of HRV and BRS was planned. In the first follow-up, there was a further reduction in HRV measures and completely diminished BRS (Table 1). He had sudden death before the scheduled 2nd follow-up visit. The death occurred at home at about 1:30 AM. He visited a physician about 4 hours before his death, no neurological or any other specific abnormalities were found at the time of examination.

**Table 1:** Heart rate variability and baroreflex sensitivity of the patient.

Parameters	First visit	Follow-up visit after 3 months	Reference values1 Mean±SD
Time domain measures of HRV^*^^†^			
SDNN (ms)	11.75	141±39	
RMSSD^‡^	3.99	2.39	27±12
pNN50^§^ (%)	0	0	
Frequency domain measures of HRV			
LF Power^||^ (ms^2^)	10.3	5.11	1170±416
HF Power (ms^2^)	1.47	0.37	975±203
Total Power (ms^2^)	89.74	20.35	3466±1018
BRS^**^, ms/mmHg	0	0	14±9 (mean±SD)7

HRV* = Heart rate variability,

SDNN† = Standard deviation of R-R intervals,

**RMSSD‡:** = root mean square of successive R-R intervals differences,

**pNN50§:** = percentage of the number of R-R intervals differences > 50 ms,

LF|| = low frequency, HF, high frequency and

BRS** = baroreflex sensitivity.

## DISCUSSION

The present case had angina on exertion (NYHA II). However, the thorough investigation showed no evidence of IHD, pleuritis, or indigestion, also showed no evidence of diabetes mellitus, hypertension, kidney diseases, hyperlipidemia, or other known disorders except the rise in ESR. However, there was no evidence of any inflammatory diseases or infections.

Both HRV and BRS were markedly reduced. In HRV; low frequency (LF) power, the marker of both cardiac sympathetic and parasympathetic tones, and high frequency (HF) power, the marker of cardiac parasympathetic tone^1^ was markedly reduced. Time domain measures of HRV; SDNN, RMSSD and pNN50; markers of cardiac parasympathetic tone,^1^ were also markedly reduced. These reports indicate a marked reduction in overall cardiac autonomic modulation. The completely diminished BRS indicates loss of cardiovascular autonomic reflex integrity.^5^

Such types of reduction both in HRV and BRS are reported in acute MI patients,^1–6^ however, the present case had no evidence of MI. He had normal 12-lead ECG, TMT and echocardiogram findings. The Tl-201 MPS also showed no evidence of cardiac stress-induced ischemia or fixed perfusion defects or any wall motion abnormality or any cardiac micro-vasculature abnormality. Ejection fraction and rate pressure products were within normal limits.

It was evidenced that the present case was not the case of syndrome X, who presents with symptoms of angina.^8^ With positive TMT and microvasculature abnormality, but normal angiography. In the present case, both TMT and Tl-201 MPS were normal. Thus, angiography was not recommended to be performed following the guidelines for the diagnosis and management of patients with stable IHD.^9^

The patient's attitude towards life was positive as assessed with the WHO scoring system.^6^ Unfortunately, the patient died within 6 months of follow-up. The death occurred at home at about 1:30 AM. He visited a physician about 4 hours before death when he felt uneasiness. On examination, no neurological or any other specific abnormalities were found. Suddenly the death occurred at night.

There are many potential life-threatening channelopathies such as Brugada syndrome, long QT, short QT, catecholaminergic polymorphic VT, arrhythmogenic right ventricular dysplasia (ARVD), early repolarization. The 12-leads ECG tracing was examined thoroughly no above-mentioned abnormalities were found in the present case. Neither any abnormality was found in ECG recorded during TMT and 24-hours Holter recording. Variant angina is also one of the risk factors of sudden death. Clinically, there was no sign and symptoms of variant angina in the patient. The 24-h Holter recording also showed no ischemic episodes when the patient experienced symptoms of angina.

The patient had a low total cholesterol level. Low total cholesterol level was also found associated with mortality in coronary heart disease,^10^ stroke, heart failure and cancer patients.^11^ In the present case, these diseases were not evidenced.

The mechanism of sudden death has ever been an enigma to medicine. The mechanisms of sudden death in the patients of epilepsy (sudden unexplained death in epileptic patients, SUDEP) and infants (sudden infant death syndrome, SIDS) have been investigated at length. Reasons point towards the possibility of autonomic mediation. The exact mechanism is not known. In the present case also there is evidence of markedly diminished cardiac autonomic tone and cardiovascular autonomic reflex integrity indicating autonomic mediation. In addition to this low total cholesterol level in the patient is an additional component. The relationship between low total cholesterol and HRV is not known.

In conclusion, the reported case presented with typical chest pain as angina. However, a thorough investigation showed no evidence of any cardiac or other systemic diseases except marked reduction in cardiac autonomic modulation as assessed with short-term HRV and cardiovascular autonomic reflex integrity as assessed with spontaneous BRS. Unexpectedly, the patient had sudden death within 6 months of follow up. This case report provides remarkable information about a risk of sudden death in a patient presenting with chest pain as angina in the absence of any known systemic disease. It may be useful to both clinicians and cardiovascular researchers.

Several patients in the community present with typical chest pain as angina. However, in an investigation, they have no cardiovascular or other known diseases that mimic angina. It is not known whether these patients have cardiac dysautonomia and the risk of sudden death. This case report may draw the attention of clinicians and cardiovascular researchers to them.
